# Tuberculosis associated factors caused by *Mycobacterium tuberculosis* of the RD^Rio^ genotype

**DOI:** 10.1590/0074-02760160347

**Published:** 2017-02-16

**Authors:** Eloise Brasil Moraes, Letícia Slompo, Amanda Juliane Finardi, Heloisa Paro Pedro da Silveira, Luciana Ruiz, Harrison Magdinier Gomes, Virginia Bodelão Richini, Philip Suffys, Carlos Magno Castelo Branco Fortaleza, Ricardo Cavalcanti, Ida Maria Foschiani Dias Baptista

**Affiliations:** 1 Instituto Lauro de Souza Lima, Divisão de Pesquisa e Ensino, Equipe Técnica de Microbiologia, Bauru, SP, Brasil; 2 Universidade Estadual Paulista Júlio de Mesquita Filho, Faculdade de Medicina de Botucatu, Doenças Tropicais, Botucatu, SP, Brasil; 3 Instituto Adolfo Lutz, Centro de Laboratório Regional de São José do Rio Preto, Núcleo de Ciências Biomédicas, São José do Rio Preto, SP, Brasil; 4 Instituto Adolfo Lutz, Centro de Laboratório Regional de Bauru, Núcleo de Ciências Biomédicas, Bauru, SP, Brasil; 5 Fundação Oswaldo Cruz-Fiocruz, Laboratório de Biologia Molecular aplicada a Micobactérias, Rio de Janeiro, RJ, Brasil

**Keywords:** Mycobacterium tuberculosis, RD^Rio^, tuberculosis, genotyping, epidemiology

## Abstract

**BACKGROUND:**

Tuberculosis (TB) continues to be a disease that affects many countries around the world, including Brazil. Recently, a subtype of Latin American-Mediterranean family strain was identified and characterised by RD^Rio^. The strain has been associated with different characteristics of the disease.

**OBJECTIVES:**

In the present study we investigated the association of epidemiological, clinical, radiological and bacteriological variables with pulmonary tuberculosis caused by RD^Rio^ *Mycobacterium tuberculosis* strain in large regions of São Paulo.

**METHODS:**

We conducted a cross-sectional study in 530 patients with pulmonary tuberculosis, diagnosed using sputum culture, from two regions of the São Paulo state in Brazil. The samples were brought to São Paulo reference laboratories for epidemiological, clinical, radiological and bacteriological analyses, and the data were obtained from a TB notification system. RD^Rio^ genotyping and Spoligotyping of the samples were performed. For the analysis of the categorical variables we used the chi-square test or the Fisher’s exact test, and for the continuous variables, the Mann-Whitney test. In addition, a logistic regression was used for multivariate analysis. Differences with p < 0.05 were considered significant.

**FINDINGS The RD^Rio^:**

deletion was identified in 152 (28.7%) samples. In the univariate analysis, both the age groups above 25 years and alcohol consumption were associated with the RD^Rio^ deletion. The multivariate analysis confirmed the association of the RD^Rio^ deletion with the age groups: 25-35 years old [OR: 2.28 (1.02-5.07; p = 0.04)] and 36-60 years old (OR: 2.36 (1.11-5.05); p = 0.03], and also with alcohol consumption [OR: 1.63 (1.05-2.54); p = 0,03].

**MAIN CONCLUSIONS:**

In this study, we identified new factors associated with the *M. tuberculosis* of the RD^Rio^ deletion strains infection.

Tuberculosis (TB) is an infectious disease that kills nearly 2 million people each year. The disease emerged about 70,000 years ago, with the migration of anatomically modern humans from Africa, and expanded as a result of the increase in human population density during the Neolithic (Comas et al. 2013). TB continues to be a global epidemic, and if left untreated, has a mortality rate of ~70% in people with positive sputum smears (Fogel 2015).

Although the incidence of tuberculosis has decreased in recent years, 9.6 million new cases and 1.5 million deaths were reported worldwide in 2014 (WHO 2014). Brazil is among the 22 countries with the highest TB incidence worldwide. The majority of the cases reported in Brazil were concentrated in the Southeast, where the state of São Paulo (SP) accounted for 20% of the disease incidence in the country (Wysochi et al. 2016).

Molecular typing, based on genetic markers, allows the rapid identification of *Mycobacterium tuberculosis* complex strains (MTC) and provides useful tools for examining the transmission and the development of the mycobacteria (Sola et al. 2001, Brosch et al. 2002, Gagneux et al. 2006). A polymerase chain reaction (PCR) identification based on the amplification of multiple deleted loci can distinguish between the MTC species (Huard et al. 2003).

Lazzarini et al. (2007) reported that MTC isolates, designated as RD^Rio^, were found to contain a chromosomal deletion of more than 26 kb. The authors reported that further analysis of the RD^Rio^ strains with a spoligotyping molecular technique confirmed that all isolates belong to Latin American-Mediterranean family (LAM). The presence of RD^Rio^ *M. tuberculosis* isolates have been reported in at least 18 other countries in Europe, Africa, and the Americas, in which all the isolates belonged to the LAM genotype of the Euro-American lineage (Gibson et al. 2008, Weisenberg et al. 2012). The RD^Rio^ *M. tuberculosis* is the most common cause of TB in Rio de Janeiro and other regions of Brazil (Lazzarini et al. 2008, Oelemann et al. 2011).

Recent studies suggest that *M. tuberculosis* strains are well adapted to human populations and that they are older and have a greater genetic diversity than previously thought (Alix et al. 2006). This diversity may have an important effect in different clinical and epidemiological aspects of tuberculosis. However, the current knowledge about the influence of TB strain diversity on the development of the disease is still scarce (Barbosa et al. 2012). Considering this, in the present study we investigated the association of epidemiological, clinical, radiological and bacteriological variables with pulmonary tuberculosis caused by RD^Rio^ *M. tuberculosis* strains in large regions of São Paulo.

## PATIENTS AND METHODS

A cross-sectional population-based study was conducted on pulmonary tuberculosis cases reported in the period 2012-2014. Two regions of the state of São Paulo were considered for this study: 69 municipalities belonging to the Regional Health Department (DRS) Section VI (Bauru) and 102 municipalities of the DRS Section XV (São José do Rio Preto).

A total of 530 tuberculosis patients with positive sputum cultures for *M. tuberculosis* were recruited. Since the detection of the RD^Rio^ deletion is only possible using the isolated culture, patients without sputum culture were not included in the study. The patients’ data were obtained through the Notification System and Monitoring of Tuberculosis Cases (TB-WEB) of the State Department of Health from the state of São Paulo - Brazil (SES/CCD/CVE). The epidemiological, clinical, radiological and bacteriological data for each patient were filled in the TB-WEB system for the epidemiological surveillance in each municipality.

*M. tuberculosis culture samples* - The *M. tuberculosis* culture samples were collected in the laboratories of the Adolfo Lutz Institute from both DRS Sections (VI and XV) and brought to the Instituto Lauro de Souza Lima for analysis. Biochemical characterisation and multidrug-resistance tests (INH, RMP, SM, and EMB) were performed as recommended by the Ministry of Health of Brazil. The susceptibility of the samples to PZA was tested separately by means of the pyrazinamidase assay (MS/SVS/DVE 2008).

*Multiplex PCR and Spoligotyping of RD* ^*Rio*^ *strains* - The identification of RD^Rio^ deletions was performed as described by Lazzarini et al. (2007) and Gibson et al. (2008). The RD^Rio^ and wild type (WT) genotypes were identified by the detection of the 1175 and 530 bp standard bands, respectively. Sample spoligotyping was performed according to the method reported by Kamerbeek et al. (1997). The spoligotype patterns were recorded in an octagonal code and in a 43-digit binary format, representing 43 spacers (Filliol et al. 2002). The *M. tuberculosis* patterns were compared with the SpolDB4 database (Brudey et al. 2006) of the Pasteur Institute in Guadeloupe (http://www.pasteur-guadeloupe.fr:8081/SITVITDemo) for type classification.

*Statistical analysis* - For the bivariate evaluation of categorical variables we used the chi-square test or the Fisher’s exact test, whereas for continuous variables analyses, the Mann-Whitney test was performed using the Epi Info version 3.5.4. These methods were applied to compare the results of the regions of Bauru (DRS-VI) and São José do Rio Preto (DRS-XV). In addition, a multivariate analysis with a logistic regression function was used to investigate the association between the presence of the RDRio marker deletion and the epidemiological variables.

Variable selection for the multivariate analysis was performed following a hierarchical procedure. Initially, the demographics were considered as distal variables. The variables that were significant (p < 0.05) were maintained and analysed in conjunction with the comorbidities and lifestyle variables, which were considered as intermediate variables. The variables which in this analysis resulted in a p < 0.05 were retained and further analysed together with the proximal variables (clinical, bacteriological and radiological). For the logistic regression, the selected variables were presented in terms of the odds ratios and their 95% confidence interval. This analysis was performed using the SPSS software version 20 considering significant variables for p < 0.05.

## RESULTS

In the period covered by this study, 530 patients with positive sputum cultures for tuberculosis were reported: 176 from Bauru and 354 from São José do Rio Preto. Because this study used secondary data, it was not possible to obtain all the information from all the patients. The epidemiological, clinical, radiological and bacteriological data of the patients of both regions are described in Table I.

**TABLE I:** Epidemiological, clinical, radiological and bacteriological data of 530 tuberculosis patients from two regions of the state of São Paulo, Brazil

Male	Bauru (DRS-VI)	São José do Rio Preto (DRS-XV)	p value

	130/176 (82,8)	281/354 (80,1)	0,54
Age (years)	35,0 (19,0 - 74,0)	37,0 (14,0 - 80,0)	0,05
Ethnicity			
white	79/137 (57,7)	195/314 (62,1)	0,43
black	22/137 (16,1)	21/314 (6,7)	0,003
brown	35/137 (25,5)	94/314 (29,9)	0,40
indian	1/137 (0,7)	0/314 (0,0)	0,30
yellow	0/137 (0,0)	4/314 (1,3)	0,23
Schooling			
unlettered	4/106 (3,8)	12/306 (3,9)	0,60
1 - 3 years	13/106 (12,3)	39/306 (12,7)	0,96
4 - 7 years	47/106 (44,3)	133/306 (43,5)	0,96
8 - 11 years	29/106 (27,4)	109/306 (35,6)	0,15
12 - 14 years	2/106 (1,9)	8/306 (2,6)	0,50
Comorbidities			
diabetes mellitus	8/128 (6,3)	22/329 (6,7)	0,96
HIV infection	12/125 (9,6)	56/324 (17,3)	0,06
alcoholism	32/129 (24,8)	96/329 (29,2)	0,41
smoking	17/128 (13,3)	102/329 (31,0)	0,0001
drogadiction	42/129 (32,6)	88/324 (27,2)	0,30
mental disease	1/90 (1,1)	8/323 (2,5)	0,38
Prisoner	34/109 (31,2)	94/228 (41,2)	0,09
History of tuberculosis	34/132 (25,8)	69/328 (21,0)	0,32
Tuberculosis forms			
pulmonary	164/165 (99,4)	311/328 (94,8)	0,02
extrapulmonary	1/165 (0,6)	16/328 (4,9)	0,02
Positive sputum smear	96/134 (71,6)	198/310 (63,9)	0,13
Chest X-ray			
normal	7/93 (7,5)	24/222 (10,8)	0,49
cavitary	23/93 (24,7)	74/222 (33,3)	0,16
suggestive of tuberculosis	63/93 (67,7)	124/222 (55,9)	0,06
Treatment abandonment	23/126 (18,3)	24/325 (7,4)	0,001
Deaths (all causes)	7/126 (5,6)	17/325 (5,2)	0,92

The analysis resulted in a higher prevalence of patients of African descent (16.1 vs. 6.7%; p = 0.003) with pulmonary tuberculosis only (99.4 vs. 94.8%; p = 0.02) and a higher rate of treatment abandonment (18.3 vs. 7.4%; p = 0.001) in the DRS-VI Bauru region. On the other hand, smokers (31.0 vs. 13.3%; p = 0.0001) prevailed in the cases detected in the municipalities of the DRS-XV São José do Rio Preto region.

The sensitivity to antituberculosis drugs is shown in Table II. The incidence of drug resistance was higher in the DRS-VI Bauru region (20.4 vs. 10.2%; p = 0.08), with significant data for pyrazinamide resistance (50.0 vs. 4.7; p = 0.02).

**TABLE II:** Drug resistance in tuberculosis patients from two regions of the state of São Paulo, Brazil

No. of patients with resistance	Bauru	São José do Rio Preto	p value

	4/44 (9,1)	22/314 (7,0)	0,39
No. total of identified resistance	9/44 (20,4)	32/314 (10,2)	0,08
No. drug resistance			
Rifampicin	2/43 (4,7)	6/313 (1,9)	0,24
Isoniazid	3/41 (7,3)	10/314 (3,2)	0,18
Pyrazinamide	2/4 (50,0)	3/64 (4,7)	0,02
Ethambutol	0/32 (0,0)	0/240 (0,0)	---
Streptomycin	2/33 (6,1)	13/238 (5,5)	0,56

The RD^Rio^ deletion was identified in 152 (28.7%) culture samples. The analysis of the factors associated with the presence of this deletion was analysed in 429 patients only. The rest of the patients were not admitted in the analysis for the lack of data. In the univariate analysis, the age groups above 25 years and alcohol-using patients were associated with the presence of the RD^Rio^ deletion, while a history of tuberculosis and drug resistance were marginally significant (Table III).

**TABLE III:** Univariate analysis of the epidemiological, clinical, microbiological and radiological factors tassociated with the presence of RDRio deletion in 429 patients with tuberculosis

Male	Deletion RD^Rio^		Odds ratio (CI 95%)	p value

	Presence nº patients (%)	Absence nº patients (%)		
	
	127 (83,6)	302 (79,9)	1,27 (0,76 - 2,19)	0,19
Age group				
under 24 years*	9 (5,9)	53 (14,0)	---	---
25 - 35 years	43 (28,3)	101 (26,7)	2,50 (1,13 - 5,53)	0,03
36 - 60 years	89 (58,6)	202 (53,4)	2,59 (1,22 - 5,48)	0,01
over 60 years	11 (7,2)	22 (5,8)	2,94 (1,07 - 8,09)	0,03
Ethnicity				
white*	79 (52,0)	195 (51,6)	---	---
brown	35 (23,0)	94 (24,9)	0,91 (0,57 - 1,46)	0,72
black	10 (6,6)	33 (8,7)	0,74 (0,35 - 1,59)	0,45
others	1 (0,7)	4 (1,1)	0,61 (0,06 - 5,60)	0,66
Schooling				
1 - 3 years*	15 (9,9)	37 (9,8)	---	---
4 - 7 years	57 (37,5)	123 (32,5)	1,14 (0,58 - 2,24)	0,69
8 - 11 years	36 (23,7)	102 (27,0)	0,87 (0,42 - 1,77)	0,70
12 - 14 years	1 (0,7)	9 (2,4)	0,24 (0,03 - 2,35)	0,23
over - years	0 (0,0)	5 (1,3)	---	0,97
unlettered	5 (3,3)	11 (2,9)	1,12 (0,33 - 3,78)	0,85
Prisoner	32 (21,1)	96 (25,4)	0,78 (0,48 - 1,25)	0,17
Health professional	3 (2,0)	2 (0,5)	3,77 (0,42 - 45,60)	0,14
HIV	18 (11,8)	50 (13,2)	0,88 (0,46 - 1,60)	0,39
Diabetes	7 (4,6)	23 (6,1)	0,74 (0,26 - 1,84)	0,33
Alcoholism	46 (30,3)	82 (21,7)	1,56 (1,02 - 2,38)	0,02
Smoking	35 (23,0)	84 (22,2)	1,04 (0,64 - 1,67)	0,46
Mental disease	3 (2,0)	6 (1,6)	1,24 (0,19 - 5,93)	0,50
Previous history of tuberculosis	23 (17,6)	80 (24,3)	0,66 (0,37 - 1,13)	0,07
Year of diagnosis				
2012*	23 (19,7)	72 (26,1)	---	---
2013	50 (42,7)	102 (37,0)	1,49 (0,83 - 2,65)	0,17
2014	44 (37,6)	102 (37,0)	1,25 (0,73 - 2,15)	0,40
Pulmonary tuberculosis	146 (96,1)	365 (96,6)	0,86 (0,30 - 2,83)	0,47
Positive sputum smear	94 (61,8)	250 (66,1)	0,83 (0,55 - 1,25)	0,20
Cavitary	24 (15,8)	73 (19,3)	0,78 (0,45 - 1,32)	0,20
Drug resistance	4 (2,6)	22 (5,8)	0,43 (0,10 - 1,32)	0,08

CI 95%: confidence interval of 95%; *: category of reference.

The multivariate analysis confirmed the association between RD^Rio^ deletion and the 25-35 years old age group [OR: 2.28 (1.02 to 5.07)] and the 36-60 year old age group [OR: 2.36 (1.11 to 5.05)], as well as with elitism [OR: 1.63 (1.05 to 2.54)], Table IV. Spoligotyping results were unsatisfactory due to problems with the membranes used for sample hybridization. Based on the spoligotype analysis, 21 RD^Rio^ isolates belonged to the families LAM 1, LAM 2, LAM 3, LAM 4, LAM 5, and LAM 9, whereas the rest of the isolates were typified within the U H1, T1 and Unknown families.

**TABLE IV:** Multivariate analysis of the epidemiological, clinical, microbiological and radiological factors associated with the presence of RDRio deletion in 429 patients with tuberculosis

Age group	Odds ratio (CI 95%)	p value
under 24 years*	---	---
25 - 35 years	2,28 (1,02 - 5,07)	0,04
36 - 60 years	2,36 (1,11 - 5,05)	0,03
over 60 years	2,69 (0,96 - 7,52)	0,06
Alcoholism	1,63 (1,05 - 2,54)	0,03

CI 95%: confidence interval of 95%; *: category of reference.

## DISCUSSION

TB is a global disease that affects not only individual patients, but also the community. Tuberculosis control goes beyond conventional strategies, therefore it is necessary to understand the interaction of the disease with the social and economic culture (Eufrásio et al. 2016). *M. tuberculosis* has a clonal population structure and until recently, it has been regarded to have little genetic variation (Sreevatsan et al. 1997, Hirsh et al. 2004). However, studies examining *M. tuberculosis* isolates from broader geographical distributions via whole-genome scanning revealed a cladistics phylogeographical distribution with significant variation between the main lines, each associated with a specific geographical region (Gagneux & Small 2007, Caws et al. 2008).

Several studies have used genotyping assays to characterise the population structure of *M. tuberculosis* and its transmission in defined communities. The high prevalence of TB caused by identical or related strains in a community may be caused by recent introduction of isolates, increased of its virulence, or even epidemiological factors that facilitate TB transmission. However, it is still unknown how the genetic makeup of *M. tuberculosis* isolates determines the transmission or the severity of the disease, as only a few studies have reported this type of analysis (Coscolla & Gagneux 2010, Vinhas et. al. 2013).

Lazzarini et al. (2007) described an *M. tuberculosis* strain belonging to the LAM family. The strain (RD^Rio^) had a large 26.3-Kb deletion (Long Sequence Polymorphism - LSP), which included 10 genes, in its DNA sequence.

The prevalence of *M. tuberculosis* samples with an RD^Rio^ deletion varied with the geographical location. In this study, we found this behaviour in 28.7% of the samples. These results were similar to those reported by Barbosa et al. (2012) in the city of Rio de Janeiro, in which the strain was detected in 26.5% of the cases (Barbosa et al. 2012). The occurrence of the RD^Rio^ deletion in the samples was lower than that found in Belo Horizonte (37%) and in Porto Alegre (38%).

Patients in both the regions analysed in this study, DRS-VI Bauru and DRS-XV São José do Rio Preto, showed some differences. There was a higher prevalence of patients of African descent, patients with only pulmonary tuberculosis, and higher treatment-dropout rate in DRS-VI Bauru, while smokers prevailed in the municipalities of DRS-XV São José do Rio Preto. Apparently, these differences did not have an impact on the presence or absence of the RD^Rio^ deletion, and they may be related to the geographical characteristics of every region. In general, the strains were not drug resistant, with the DRS-VI Bauru samples possessing a greater drug resistant capacity. The resistance of the strains to pyrazinamide was higher in the latter region. However, since the number of samples was very small, this result may not be representative of the local reality.

The age groups 25-35 and 36-60 years old were associated with the presence of RD^Rio^ deletion, indicating that these strains are less infectious in young individuals under 25 years old and in patients over 60 years old. In previous studies, no positive association was found between age and the presence of RD^Rio^ deletion (Lazzarini et al. 2007, Gibson et al. 2008, Barbosa et al. 2012, Weisenberg et al. 2012). The discrepancy on these results may be attributed to the fact that the latter studies considered the average or the median values rather than the age.

Considering the multivariate analysis, a positive association was found between alcohol consumption and the presence of RD^Rio^ deletion. The former condition increased 1.6 times the chance of *M. tuberculosis* disease by this strain. A couple of studies have assessed the effect of alcohol consumption on the disease and found no positive association (Gibson et al. 2008, Dalla Costa et al. 2013).

Although we did not assess the nutritional status of the patients due to a lack of data, it is possible that the association of the disease with alcohol consumption may reflect the fact that an impaired nutritional status and a poor immune system favour *M. tuberculosis* infection. Lazzarini et al. (2007) found an association between weight loss and the presence of RD^Rio^ deletion, which supports the critical effect of malnutrition on the disease. There is evidence that the RD^Rio^ deletion strains are associated with an increased transmissibility of tuberculosis bacillus (Lazzarini et al. 2007, Weisenberg et al. 2012). In general, alcoholic patients live in unhealthy and crowded environments and this could also explain this association. However, we did not evaluate the association of the socioeconomic conditions of the patients with alcohol consumption.

Previous studies have reported additional positive associations with RD^Rio^ strains carrying the deletion. In a prospective study comprising 105 patients, Lazzarini et al. (2008) found 8.9 times more lung cavitation cases in patients infected by RD^Rio^ deletion strains. We were not able to prove this association because with the secondary data used in this study we could not confirm the occurrence of cavitation.

In this study, no association was found between antituberculosis-drug resistance and RD^Rio^ deletion. This can be explained by the low resistance prevalence in the studied strains. A Brazilian study conducted in Porto Alegre reported an RD^Rio^ association with drug resistance (Dalla Costa et al. 2013). In the present study, 99% of the samples with deletions were resistant, while only 63% of the *M. tuberculosis* wild strains presented multidrug resistance (MDR). The samples were obtained from a referral centre for MDR TB. It is possible that this association is the result of the high rate of resistance in the population, so that it does not represent the population with TB in Brazil. A study conducted in New York City reported a 1.6-fold increase in the resistance to isoniazid in the presence of RD^Rio^ deletion (Weisenberg et. al. 2012). Further studies are needed to verify the association of resistance with the RDRio deletion strain.

Barbosa et al. (2012) evaluated 272 patients in Rio de Janeiro. Using a univariate analysis, the authors did not find an association of the infection by RD^Rio^ deletion strains with epidemiological, clinical and radiological factors. These results demonstrate the need for further studies to assess the role of this strain as the cause of tuberculosis.

The major limitation of this study was the use of secondary data. Considering the lack of important patient information regarding the epidemiological, clinical, radiological and bacteriological results, a bias must be considered in the interpretation of the associations reported in this work.

Finally, this study contributes to the knowledge of the association between infection by *M. tuberculosis* strains with RD^Rio^ deletion and the 25-60 years-old age groups and/or alcohol consumption. Future studies with larger sample sizes are needed to better understand these associations.

## References

[B1] Alix E, Godreuil S, Blanc-Potard AB (2006). Identification of a Haarlem genotype-specific single nucleotide polymorphism in the mgtC virulence gene of Mycobacterium tuberculosis. J Clin Microbiol.

[B2] Barbosa CB, Lazzarini LCO, Elias AR, Leung JAM, Ribeiro SB, Silva MG (2012). Tuberculosis caused by RD^Rio^Mycobacterium tuberculosis is not associated with differential clinical features. Int J Tuberc Lung Dis.

[B3] Brosch R, Gordon SV, Marmiesse M, Brodin P, Buchrieser C, Eiglmeier K (2002). A new evolutionary scenario for the Mycobacterium tuberculosis complex. Proc Natl Acad Sci USA.

[B4] Brudey K, Driscoll JR, Rigouts L, Prodinger WM, Gori A, Al-Hajoj SA (2006). Mycobacterium tuberculosis complex genetic diversity: mining the fourth international spoligotyping database (SpolDB4) for classification, population genetics and epidemiology. BMC Microbiol.

[B5] Caws M, Thwaites G, Dunstan S, Hawn TR, Lan NT, Thuong NT (2008). The influence of host and bacterial genotype on the development of disseminated disease with Mycobacterium tuberculosis. PLoS Pathog.

[B6] Comas I, Coscolla M, Luo T, Borrell S, Holt KE, Kato-Maeda M (2013). Out-of-Africa migration and Neolithic coexpansion of Mycobacterium tuberculosis with modern humans. Nat Genet.

[B7] Coscolla M, Gagneux S (2010). Does M. tuberculosis genomic diversity explain disease diversity?. Drug Discov Today Dis Mech.

[B8] Dalla Costa ER, Lazzarini LC, Perizzolo PF, Díaz CA, Spies FS, Costa LL (2013). Mycobacterium tuberculosis of the RD^Rio^ genotype is the predominant cause of tuberculosis and associated with multidrug resistance in Porto Alegre City, South Brazil. J Clin Microbiol.

[B9] Eufrásio R, Alcobia M, Correia L (2016). Pulmonary tuberculosis epidemiology in Coimbra’s District (2000-2011): information is essential tounder stand high risk groups. Rev Port Pneumol.

[B10] Filliol I, Driscoll JR, van Soolingen D, Kreiswirth BN, Kremer K, Valétudie G (2002). Global distribution of Mycobacterium tuberculosis spoligotypes. Emerg Infect Dis.

[B11] Fogel N (2015). Tuberculosis: a disease without boundaries. Tuberculosis (Edinb).

[B12] Gagneux S, DeRiemer K, Van T, Kato-Maeda M, Jong BC, Narayanan S (2006). Variable host-pathogen compatibility in Mycobacterium tuberculosis. Proc Natl Acad Sci USA.

[B13] Gagneux S, Small PM (2007). Global phylogeography of Mycobacterium tuberculosis and implications for tuberculosis product development. Lancet Infect Dis.

[B14] Gibson AL, Huard RC, van Pittius NCG, Lazzarini LCO, Driscoll J, Kurepina N (2008). Application of sensitive and specific molecular methods to uncover global dissemination of the major RDRio sublineage of the Latin American-Mediterranean Mycobacterium tuberculosis spoligotype family. J Clin Microbiol.

[B15] Hirsh AE, Tsolaki AG, DeRiemer K, Feldman MW, Small PM (2004). Stable association between strains of Mycobacterium tuberculosis and their human host populations. Proc Natl Acad Sci USA.

[B16] Huard RC, Lazzarini LCO, Butler WR, van Soolingen D, Ho JL (2003). PCR-based method to differentiate the subspecies of the Mycobacterium tuberculosis complex on the basis of genomic deletions. J Clin Microbiol.

[B17] Lazzarini LC, Huard RC, Boechat NL, Gomes HM, Oelemann MC, Kurepina NE (2007). Discovery of a novel Mycobacterium tuberculosis lineage that is a major cause of tuberculosis in Rio de Janeiro, Brazil. J Clin Microbiol.

[B18] Lazzarini LC, Spindola SM, Bang H, Gibson AL, Weisenberg S, Carvalho WS (2008). RD^Rio^Mycobacterium tuberculosis infection is associated with a higher frequency of cavitary pulmonary disease. J Clin Microbiol.

[B19] Ministério da Saúde, Secretaria de Vigilância em Saúde, Departamento de Vigilância Epidemiológica (2008). Manual nacional de vigilância laboratorial da tuberculose e outras micobactérias.

[B20] Oelemann MC, Gomes HM, Willery E, Possuelo L, Lima KVB, Allix-Béguec C (2011). The forest behind the tree: phylogenetic exploration of a dominant Mycobacterium tuberculosis strain lineage from a high tuberculosis burden country. PLoS ONE.

[B21] Sola C, Filliol I, Legrand E, Mokrousov I, Rastogi N (2001). Mycobacterium tuberculosis phylogeny reconstruction based on combined numerical analysis with IS1081, IS6110, VNTR, and DR-based spoligotyping suggests the existence of two new phylogeographical clades. J Mol Evol.

[B22] Sreevatsan S, Pan X, Stockbauer KE, Connell ND, Kreiswirth BN, Whittam TS (1997). Restricted structural gene polymorphism in the Mycobacterium tuberculosis complex indicates evolutionarily recent global dissemination. Proc Natl Acad Sci USA.

[B23] Vinhas SA, Palaci M, Marques HS, Aguiar PPL, Ribeiro FK, Peres RL (2013). Mycobacterium tuberculosis DNA fingerprint clusters and its relationship with RD(Rio) genotype in Brazil. Tuberculosis (Edinb).

[B24] Weisenberg SA, Gibson AL, Huard RC, Kurepina N, Bang H, Lazzarini LC (2012). Distinct clinical and epidemiological features of tuberculosis in New York City caused by the RD^Rio^Mycobacterium tuberculosis sublineage. Infect Genet Evol.

[B25] WHO - World Health Organization (2014). Global Tuberculosis Report.

[B26] Wysocki AD, Villa TCS, Arakawa T, Brunello MEF, Vendramini SHF, Monroe AA (2016). Latent tuberculosis infection diagnostic and treatment cascade among contacts in primary health care in a city of São Paulo state, Brazil: cross-sectional study. PLoS ONE.

